# Bone tissue preservation in seawater environment: a preliminary comparative analysis of bones with different post-mortem histories through anthropological and radiological perspectives

**DOI:** 10.1007/s00414-021-02636-6

**Published:** 2021-08-16

**Authors:** Barbara Bertoglio, Carmelo Messina, Annalisa Cappella, Emanuela Maderna, Debora Mazzarelli, Stanilla Lucheschi, Francesco Sardanelli, Luca Maria Sconfienza, Chiarella Sforza, Cristina Cattaneo

**Affiliations:** 1 LABANOF, Dipartimento di Scienze Biomediche per la Salute, Sezione di Medicina Legale, Università degli Studi di Milano, Milan, Italy; 2 IRCCS Istituto Ortopedico Galeazzi, Milan, Italy; 3 Dipartimento di Scienze Biomediche per la Salute, Università degli Studi di Milano, Milan, Italy; 4 U.O, Laboratorio di Morfologia Umana Applicata, IRCCS Policlinico San Donato, San Donato Milanese, Milan, Italy; 5 IRCCS Policlinico San Donato, Milan, Italy; 6 Laboratorio di Anatomia Funzionale dell’Apparato Stomatognatico (LAFAS), Dipartimento di Scienze Biomediche per la Salute, Università degli Studi di Milano, Milan, Italy

**Keywords:** Taphonomy, Bone histology, Bone mineral density (BMD), Bone tissue preservation, Marine bone taphonomy

## Abstract

Bone taphonomy is a widely investigated topic; however, few data are available concerning marine bone taphonomy, especially on remains recovered from great depths and with short post-mortem intervals. To date, few studies have evaluated the bony changes which occur in seawater compared to samples with different post-mortem histories, and none through a comparative analysis of different approaches. To this purpose, this pilot study aims to examine the influence of seawater on bone preservation compared to other depositional contexts by multiple perspectives. Forty-nine human bone samples (femurs or tibiae) recovered from different environments (sea water, fresh water, outdoor, burial in coffin) were compared by macroscopic, microscopic and bone densitometric approaches. In order to investigate organic and inorganic components, undecalcified and decalcified histology of thin sections was performed. The analyses revealed a well-preserved bone tissue both macroscopically (92%) and microscopically (97% and 95% for undecalcified and decalcified sections). No significant differences were detected from radiological densitometric investigations (BMD = 1.6 g/cm^2^ ± 0.1), except between old and young individuals (p value < 0.001). Differences were observed for body decomposition and few scavenged samples (3/15). However, even if slight variations were observed, no relation was recorded with the depositional contexts. We found a similar bone preservation in the four environments at the time of recovery, both macroscopically and microscopically, but also with radiological densitometric investigations. Our observations enriched the literature on bone taphonomy, providing data on bone tissue preservation in the early post-mortem period from a multidisciplinary perspective, paving the way for further studies on the topic.

## Introduction

Bone taphonomy has always fascinated forensic scientists for many reasons, some more valid perhaps than others: at times it seems to have strong correlations, but not always, with postmortem interval (PMI), in other cases it seems to relate to the burial or deposition site of the body, and sometimes macroscopic taphonomy may be envisaged as reflecting the potential the bone has for providing microscopic, biomolecular, chemical information. Many times, however, research has proven that these correlations may not be so [see, for example, 1–5].

Since the nineteenth century, several studies have been carried out to better understand the diagenetic processes which affected human remains coming from different environments [e.g. 6–16]. Over the years, several mechanisms responsible for bone deterioration have been described, including biological and chemical degradation of the collagen fraction and dissolution and recrystallization phases of the mineral component [9, 12, 13, 16, 17]. A mutual protection was proposed as responsible for bone stability during the post-mortem period between these two fractions (the collagen and the mineral fractions), and its breaking would result in a rapid degradation of bone tissue [18]. Among the different mechanisms, the microbial degradation of bone has been one of the most investigated topics. In 1981, Hackett was the first to describe and characterise the forms of foci and tunnels [8], with several scientists after him proceeding with the study of bone tissue microstructure with a special focus on microbial activity in bones coming from different environments. The possible patterns of microbial activity that can be found in different contexts have been described, especially those related to outdoor and water environments, reporting the time at which the first signs were visible within bone tissue [see, for example, 15, 19–23]. However, despite the considerable number of studies, little is still known about marine bone taphonomy, especially for samples coming from deep sea and with a short PMI.

Considering the difficulties in setting experimental investigations in marine environments, information generally comes from forensic cases [24–27], which are focused on body preservation and macroscopic appearance of the recovered skeletal remains. Studies on bioerosion were also carried out, but analyses were limited to experimental substrates (e.g. limestone and PVC plates) [28–31]. Other bioerosion studies were carried out on pigs, but without analysing bone tissue when skeletonization was reached [32–34]. Considering bone histological investigations, data were recorded especially from archaeological material (tenth to sixteenth centuries) [20, 23, 35, 36], and only in few cases from recent human skeletal remains [19].

In the last years, methods other than microscopy were used in taphonomical research, such as chemical and radiological analyses. Among these, the analysis of bone mineral density (BMD) has been included in many studies. In particular, previous investigations focused on the changes in bone mineral content at several portions on human bones by different techniques (single photon absorptiometry, peripheral quantitative computed tomography (pQCT), and dual energy X-ray densitometry) [37–39], suggesting a possible relation between the density of bone elements or portions and their degree of environmental preservation [38, 39]. However, no studies have investigated BMD on skeletal remains coming from marine environments, nor have they evaluated whether variations in mineral content exist between different environments. In addition, no comparisons were carried out so far among bones recovered from different depositional scenarios with a multidisciplinary approach (medico-legal, anthropological and radiological).

Collecting more data on bone tissue preservation of contemporary samples would provide important insights on the diagenetic trajectory which affected skeletal remains, as well as regarding the mechanisms involved in the preservation/degradation of the bone components (collagen and mineral phase). This is even more important from environments for which little information is available, such as the sea [27, 40]. This information would have also important implications in laboratory analyses that involve the study of the bone tissue and its molecular components, such as age estimation, pathological diagnosis and genetic studies. In addition, comparisons with other samples with a different post-mortem history would allow identification of the peculiar traits which can differentiate the different burials and environmental changes acting as a fingerprinting of the diagenetic events.

The present pilot study aims to compare the state of preservation of bones recovered in seawater with bones coming from different environments (outdoor and fresh water environments and buried in coffin) from a macroscopic, microscopic and bone densitometric perspectives. This is the first type of comparative study in this sense to our knowledge.

## Materials and methods

### Samples

The study was conducted overall on 49 samples coming from four different contexts.

Fifteen samples (samples SW 1–15) were selected from remains recovered in a boat which had sunk in the Mediterranean Sea 100 km north of the Libyan coast and 200 km south of the Italian island of Lampedusa at a depth of about 400 m. The bodies, who laid both outside the boat (in the sea bottom) and inside cargo and deck, were recovered in different periods after the disaster (from 4 to 14 months).

Fourteen samples were selected from forensic cases, accordingly to the availability of skeletons with PMI comparable to those of the marine group. Among these, three samples were collected from individuals recovered in fresh water environments (FW 1–3), while the remaining were all recovered in outdoor environments (i.e. on the ground surface) (OE 1–10), with the exception of one individual, who was buried in soil at a depth of 1 m (OE 11). All the environments were located in the North of Italy, in Piedmont, Lombardy and Emilia Romagna Region, and the remains were recovered in a time lapse between 2000 and 2019. In this area the mean annual temperature ranges between 12 and 15 °C (13.2 °C ± 1.2 °C), with mean cumulative rainfall of 939 mm (± 343.8 mm) and mean humidity of 76% (± 5.4%) (mean data from 2000 to 2019 [41]).

Ten samples (samples CM 1–10), with a much longer PMI and buried in soil, were selected from the contemporary Milan skeletal collection, “Collezione Antropologica LABANOF” (CAL, LABANOF Anthropological Collection) [42]. The bodies came from unclaimed remains of persons who died between 1990 and the 1992, buried inside coffin and placed under the soil for 15 years at the Cimitero Maggiore in Milan (Italy), until complete skeletonization.

In addition, in order to evaluate the presence of BMD age-related changes in the samples buried in coffins (cemeterial cases), bone densitometric analyses were performed on ten complete femurs selected from ten younger individuals of the same skeletal collection (5 males and 5 females, burial in coffin, PMI: 20 years), with known ages that were comparable to the outdoor sample (age range: 20–45 years old). To this purpose, CM samples were called CM_old and CM_young, respectively.

The remains of these individuals can be used for scientific studies, in accordance with the Police Mortuary Rules (DPR 09.10.1990 n° 285, art. 43) and the Regio Decreto (08.31.1933 n° 1592, art. 32).

Femurs or tibiae were chosen for the analyses, since these bone elements were available for each case. Information on the biological profiles was acquired through demographic sources and/or anthropological analyses [43].

Details concerning each sample are reported in Table 1.

**Table 1:** Samples information. Details of the biological profiles, post-mortem interval (PMI) and environment of the samples selected for the study. For the marine environment, the section of the boat from which the victims were recovered is reported (A, recovery from the sea bottom; B, recovery from deck; C, recovery from cargo). Age at death was summarised as follows: adolescent (≤ 18 years old), young adult (19–40 years old), middle adult (41–60 years old) and elderly (> 60 years old). *ND* not determinable

Sample	Sex	Ancestry	Age	PMI	Environment	Bone sample
SW 1	Male	ND	Young adult	4 months	Sea – A	Femur
SW 2	Male	ND	Young adult	5 months	Sea – A	Tibia R
SW 3	Male	ND	Young adult	14 months	Sea – B	Femur
SW 4	Male	African	Young adult	14 months	Sea – B	Femur
SW 5	Male	ND	Young adult	14 months	Sea – B	Tibia R
SW 6	Male	ND	Young adult	14 months	Sea – B	Femur L
SW 7	Male	ND	Young adult	14 months	Sea – C	Femur L
SW 8	Male	ND	Young adult	14 months	Sea – B	Femur R
SW 9	Male	ND	Young adult	9 months	Sea – A	Femur
SW 10	Male	ND	Young adult	7 months	Sea – A	Femur R
SW 11	Male	ND	Young adult	14 months	Sea – C	Femur R
SW 12	Male	ND	Young adult	14 months	Sea – C	Tibia R
SW 13	Male	ND	Adolescent	14 months	Sea – C	Femur L
SW 14	Male	African	Young adult	7 months	Sea – A	Tibia
SW 15	Male	ND	Young adult	14 months	Sea – C	Femur
FW 1	Male	European	Young adult	< 1 year	Fresh water	Femur L
FW 2	Female	ND	Middle adult	< 6 months	Fresh water	Femur R
FW 3	Male	European	Elderly	> 1 year	Fresh water	Femur R
OE 1	Female	European	Young adult	< 6 months	Outdoor	Tibia R
OE 2	Female	African	Young adult	< 6 months	Outdoor	Femur R
OE 3	Female	European	Middle adult	< 6 months	Outdoor	Femur R
OE 4	Female	European	Adolescent	< 6 months	Outdoor	Femur R
OE 5	Male	European	Young adult	3 months	Outdoor	Femur R
OE 6	Male	European	Middle adult	1 year	Outdoor	Tibia L
OE 7	Female	European	Adolescent	3 months	Outdoor	Femur R
OE 8	Male	European	Young adult	7 months	Outdoor	Femur R
OE 9	Male	European	Young adult	3 years	Outdoor	Femur L
OE 10	Female	European	Middle adult	1 year	Outdoor	Femur R
OE 11	Female	European	Middle adult	1 months	Buried in soil	Femur R
CM 1_old	Female	European	Elderly	20 years	Buried in coffin	Tibia L
CM 2_old	Male	European	Elderly	20 years	Buried in coffin	Tibia R
CM 3_old	Male	European	Elderly	20 years	Buried in coffin	Tibia R
CM 4_old	Female	European	Elderly	20 years	Buried in coffin	Femur L
CM 5_old	Male	European	Elderly	20 years	Buried in coffin	Femur R
CM 6_old	Male	European	Elderly	20 years	Buried in coffin	Femur R
CM 7_old	Male	European	Elderly	20 years	Buried in coffin	Tibia R
CM 8_old	Female	European	Elderly	20 years	Buried in coffin	Femur L
CM 9_old	Male	European	Elderly	20 years	Buried in coffin	Femur R
CM 10_old	Female	European	Elderly	20 years	Buried in coffin	Tibia R
CM 1_young	Male	European	Young adult	20 years	Buried in coffin	Femur R
CM 2_ young	Male	European	Middle adult	20 years	Buried in coffin	Femur R
CM 3_ young	Male	European	Young adult	20 years	Buried in coffin	Femur R
CM 4_ young	Female	European	Young adult	20 years	Buried in coffin	Femur R
CM 5_ young	Female	European	Middle adult	20 years	Buried in coffin	Femur R
CM 6_ young	Female	European	Young adult	20 years	Buried in coffin	Femur R
CM 7_ young	Female	European	Middle adult	20 years	Buried in coffin	Femur R
CM 8_ young	Male	European	Young adult	20 years	Buried in coffin	Femur R
CM 9_ young	Female	European	Young adult	20 years	Buried in coffin	Femur R
CM 10_ young	Male	European	Young adult	20 years	Buried in coffin	Femur R

### Anthropological analyses

#### Body preservation

The state of decomposition of the body was evaluated as described in [25, 44]) for samples from water and outdoor/buried samples, respectively. Nine (from 1 to 9) and ten (from A to D, with additional sub-stages) categories describe the decomposition of a body from the “fresh cadaver” appearance to an early and then advanced decomposition, until the partial/complete bone exposure and final disarticulation.

To this purpose, pictures acquired during body examinations were analysed, with a special focus on the portion of the lower limbs from which samples were collected (thigh or leg).

To compare results, the scoring system defined by De Donno et al. [25] was uniformed to the four categories identified by Megyesi et al. [44] as reported in Table 2.

**Table 2:** Categories used to compare body preservation in the different environments. The first column showed the uniformed categories used for comparison. The Megyesi and De Donno’s scores corresponding to each category are also reported

Categories for comparison	Score (Megyesi et al [44])	Score (De Donno et al [25])	General description
A	A	1	Fresh cadaver
B	B	2–6	Early decomposition
C	C	7	Advanced decomposition
D	D	8–9	Skeletonization

#### Macroscopic analysis of the bone tissue

Since environments have a different influence on the macroscopic appearance of bones, the state of preservation of bone surface was evaluated using specific methods for each samples group.

Samples collected from buried remains or remains decomposed in an outdoor environment were analysed applying bone weathering stages proposed by Behrensmeyer [45]. Six stages were identified (from 0 to 5), corresponding to an increasing amount of bone alterations, such as cracking, flaking, rough area and weathered compact bone, which becomes more consistent over time. Stage 0 was associated to bone showing greasiness, no cracks or flakes and soft tissue still present, while the last phase (stage 5) corresponded to very fragile bones, easily prone to breaking.

Samples coming from the aquatic environment were evaluated according to the description reported by Pokines et al. [26]. Since no scoring system was suggested, bone appearance was described checking for the taphonomic characteristics identified by the authors in marine remains. Generally, score 0 was associated to bone showing organic sheen, fat leaching, adipocere and soft tissue still present.

#### Microscopic analysis

Two semi-circular sections were cut transversely with a hacksaw at the middle diaphysis of each bone sample (or in its proximity when it was lacking) and submitted to processing protocols for undecalcified and decalcified bone sections.

The first sections were levelled on a side using a Struers Dap-7 grinding wheel, with abrasive discs of grain size between 180 and 4000, to obtain flat and polished fragments. After the mounting of the smooth side on a glass slide with Pertex resin (Histolab Products AB), samples were grounded on the other side to get thin sections suitable for microscopic analysis.

The second sections were fixed in formalin (v/v, pH 7–7.6), ratio formalin/sample 20:1, and decalcified at room temperature with Histo-Decal, containing 14% hydrochloric acid (Histo-Line Laboratories, Milan), rinsing 1h in tap water after each step. After dehydration using an increasing alcohol series, samples were included in paraffin, cut in 5 μm sections and stained with Hematoxylin-Eosin.

Sample slides were analysed using the polarizing optical microscope Zeiss Axio Scope.A1, and images were acquired with the camera system True Chrome Hd II and the software ISCapture version 3.6.7. Bone tissue appearance was evaluated and classified according to the score system (Oxford Histological Index, OHI) described by Hedges et al. [46], as already applied by Cappella et al. and Caruso et al. [47, 48]. Six stages were identified (from 5 to 0), evaluating the amount of unaltered bone and the possibility to recognise bone structures such as osteons, lamellae and osteocyte lacunae. Bone sections with well-preserved tissue, highly similar to fresh bone were scored as “5”, while sections with less than 5% of the tissue preserved and no recognisable features were classified as “0”.

### Radiological analyses

Bone mineral density (BMD) was assessed with dual energy X-ray absorptiometry (DXA), using a Hologic QDR-Discovery W densitometer (Hologic Inc., Bedford, MA, USA). BMD areal values were expressed as g/cm^2^, which represents the bone mineral content (g) per area (cm^2^). Bone samples were scanned in 140 × 25 mm regions covering all the bone shaft available. As not all the bone specimens were complete, comparison between samples was performed on the region which resulted most common among the majority of them. In particular, since the fifteen samples from sea water environment only had a fragment sampled during autopsy examination from the middle diaphysis, this region was selected for the analysis. Only six samples lacked this part, SW 5, OE 3, OE 7, OE 10, OE 11 and CM_old 9, and were not included in the analysis.

### Statistical analysis

#### Interobserver variability

Anthropological analyses were carried out in a blind test by two anthropologists (with 5 and 10 years of experience on macro- and microscopic taphonomy) following the scoring system described in the previous paragraphs (see the Anthropological Analyses section). Interobserver agreement was calculated using the Cohen’s kappa statistic [49]. The interpretation of Cohen’s kappa was carried out as reported by Landis and Koch [50], defining values < 0 as no agreement, 0–0.20 as slight, 0.21–0.40 as fair, 0.41–0.60 as moderate, 0.61–0.80 as substantial and 0.81–1 as almost perfect agreement.

#### Mineral content variation among groups

In order to determine if the depositional environment could influence bone mineral content, statistical analyses were carried out to evaluate differences on BMD among the different groups. However, since few samples were available for the fresh water environment (3 samples) and the sample size was not comparable to the other groups, this group was not included in the statistical evaluation. Normality and homoscedasticity were verified by the Shapiro and Bartlett tests, and the comparison among group means was performed using the one-way analysis of variance (ANOVA). Post-hoc tests were made by Tukey HSD tests. Statistical significance was assessed when the p value was lower than 0.05.

Statistical analyses were carried out using the R Studio Software (version 1.2.1335) (RStudio Team (2015). RStudio: Integrated Development for R. RStudio, Inc., Boston, http://www.rstudio.com/).

## Results

### Anthropological analyses

Statistical analysis highlighted a high agreement between the two operators. Kappa values ranged between 0.70 and 0.95 (p value < 0.001), suggesting low interobserver variability (Table 3).

**Table 3:** Interobserver variation analysis. Cohen’s kappa values and the corresponding strength of agreement are reported for each analysis performed

Analysis	Cohen’s kappa	p-value	Strength of Agreement
Body preservation	0.95	< 0.001	Almost perfect
Bone preservation	0.85	< 0.001	Almost perfect
Undecalcified thin sections	0.70	< 0.001	Substantial
Decalcified thin sections	0.73	< 0.001	Substantial

Evaluation of the state of preservation of the cadavers (Table 4, section “Body decomposition”) revealed a greater skeletonization of the bodies (61% of the entire sample) and a minor early/advanced stage of decomposition in the entire sample (13% and 26%, respectively). Comparing the four environments, inter-group variability was observed. Skeletonization of the samples was recorded in all the buried cases (CM_old 1–10) and, with a low percentage, in the sea water context (67%), where other stages were still present even if in a minor amount (13% and 20% in B and C, respectively). Differently, samples coming from fresh water and outdoor environments were spread in the central (B and C) and in the last three categories (B, C, D) respectively, the latter showing the majority of bodies in the advanced stage of decomposition and skeletonization classes (46% and 36%, respectively).

**Table 4:** Body and bone preservation results. Percentages of samples from the different environments and of the entire study are reported for each category. (*SW* sea water, *FW* fresh water, *OE* outdoor environment, *CM_old* burial in coffin)

	% SW	% FW	% OE	% CM_old	% all
*Body decomposition*					
A	0	0	0	0	0
B	13	33	18	0	13
C	20	67	46	0	26
D	67	0	36	100	61
*Bone preservation*					
0-1	100	100	100	70	92
2-3	0	0	0	30	8
4-5	0	0	0	0	0
*Undecalcified thin sections*					
4-5	100	100	100	90	97
2-3	0	0	0	10	3
0-1	0	0	0	0	0
*Decalcified thin sections*					
4-5	100	100	100	80	95
2-3	0	0	0	20	5
0-1	0	0	0	0	0

**Fig. 1:**
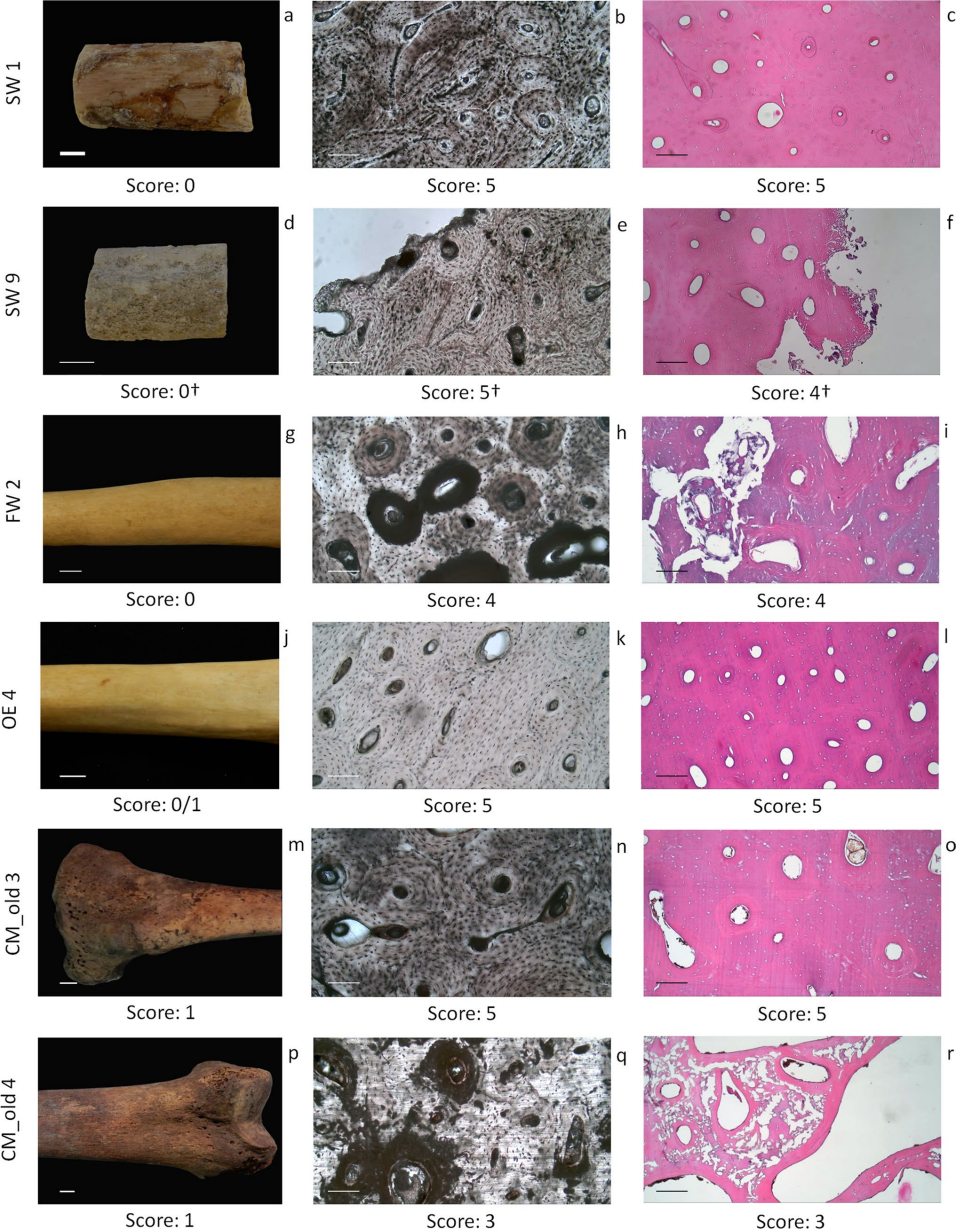
Macroscopic and microscopic appearance of representative samples for each group. First column shows macroscopic appearance of the bone shaft (**a**,**d**,**g**,**j**,**m**,**p**, scale bar: 1 cm), while the second and third columns report undecalcified (**b**,**e**,**h**,**k**,**n**,**q**) and decalcified (**c**,**f**,**i**,**l**,**o**,**r**) thin sections (100 ×, scale bar: 139.56 µm) (†: bone surface and bone thin sections showing cortical bone erosion due to marine organisms)

When bone tissue was examined, comparable results were obtained from both macroscopic and microscopic analyses (Table 4, sections “Bone preservation”, “Undecalcified thin sections” and “Decalcified thin sections”). Most of the samples were characterised by good preservation of the cortical bone tissue (92%, scores 0–1), which appeared still greasy in samples with the shortest PMI (< 36 months), and by well-preserved bone microstructure (within up to 85–95% of the tissue) in both undecalcified and decalcified thin sections (97% and 95%, respectively, scores 4–5) (Fig. 1).

No tunnels and destructive foci were detected in the samples collected from the two aquatic contexts and the outdoor environments, which showed also a similar macroscopic appearance. Cortical bone erosion related to marine organism activity was observed both macroscopically and microscopically in three samples recovered in the sea bottom 7–9 months after death (SW 9, SW 10 and SW 14). In spite of the loss of bone tissue in the external layer, the bone structure proved well-preserved inside: the medullary cavity still full of preserved bone marrow and the absence of weathering signs as well as destructive foci in the entire section represent the main findings (Fig. 1d–f). Differently, even if in a small fraction of samples (from 10 to 30%), the buried cases (CM_old cases) displayed bone weathering on the surface, mainly consisting in flaking (CM_old 6, CM_old 7 and CM_old 8), and destructive foci caused by microbial activity corresponding to circa 30% of the bone section (CM_old 1 and CM_old 4, Fig. 1q–r), while in the remaining cases the bone tissue was well-preserved (Fig. 1m– o).

**Fig. 2:**
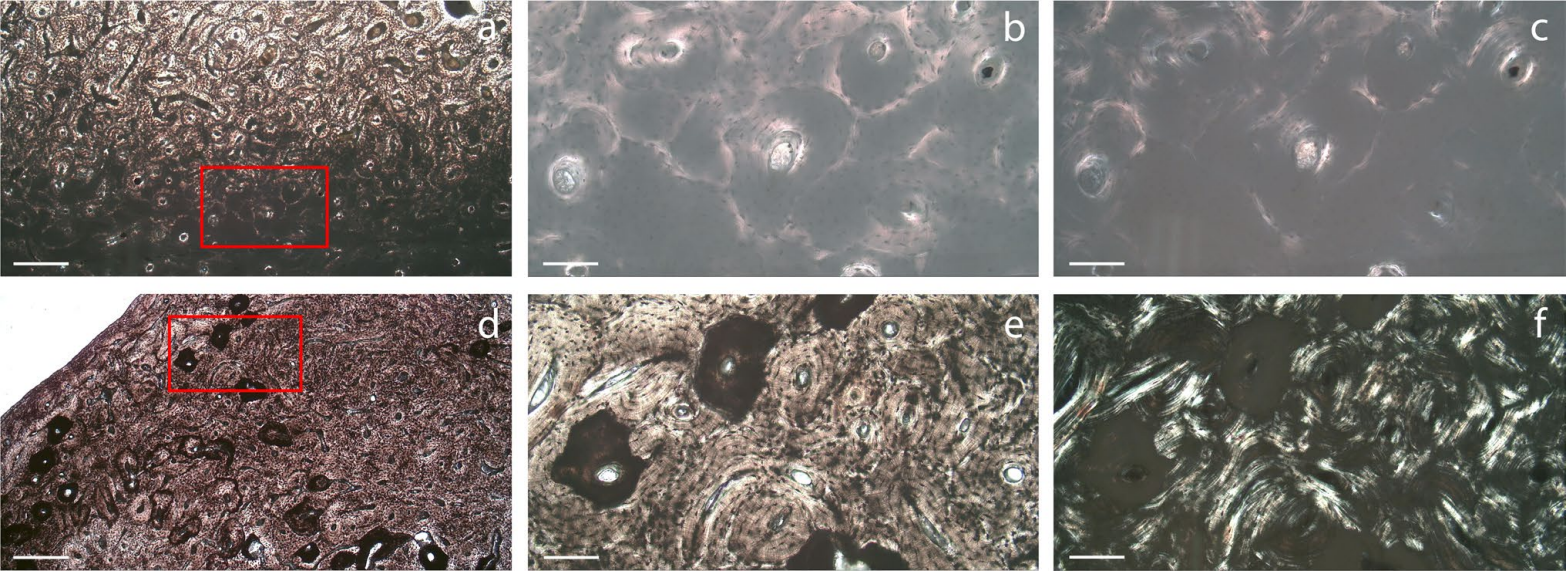
Blackish/darkened areas observed in the sea water (**a**–**c**) and fresh water (**d**–**f**) samples (**a** and **d** 25 ×, scale bar: 552.48 µm; **b** and **e** 100 ×, scale bar: 139.56 µm; **c** and **f** 100 × at polarised light, scale bar: 139.56 µm)

When attention was focused on bone tissue alterations, other than tunnels and destructive foci, blackish/darkened areas of unknown origin were identified in undecalcified thin sections of both seawater and fresh water samples. In particular, these regions were in correspondence of both external circumferential lamellae and osteons in the seawater samples SW12 and SW13 (Fig. 2a–c), and they were observed also in the middle section in fresh water samples (Fig. 2d–f). After decalcification, in the latter the same osteons appeared detached from the surrounding tissue with an appearance similar to that of insufficiently demineralised tissue (Fig. 1h–i).

**Fig. 3:**
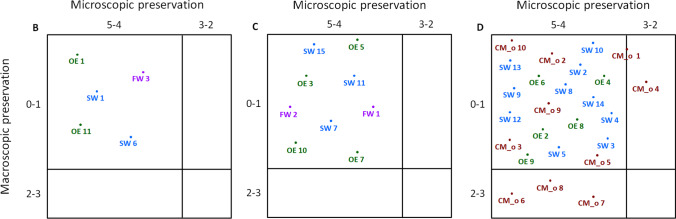
Comparison of body and bone preservation for each sample (CM_old in red, buried samples; OE in green, outdoor cases; FW in violet, fresh water samples; SW in blue, sea water samples). Big squares, namely B, C and D, correspond to body preservation staging, while rows and columns to the macroscopic and microscopic bone tissue preservation, respectively. Because the stage A for body preservation and the worst categories for macro and micro appearance (4-5 and 1-0, respectively) were not assigned, they are not shown in the figure. Sample CM_old 1 is localised between the two categories 5-4 and 3-2 due to the different score obtained analysing undecalcified and decalcified thin sections (4 and 3, respectively)

Comparing body and bone preservation, no relation was observed. In particular, complete skeletonized samples showed in the majority of the cases macroscopic and microscopic features similar to those collected from recent and early decomposed bodies (Fig. 3). In addition, the localization of most of the samples within the area characterised by well-preserved bones (Fig. 3, 0-1 and 5-4 coordinates) pointed that no relevant macroscopic and microscopic differences existed among the three different contexts.

### Radiological analyses

The BMD values obtained by the bone densitometric analyses are reported in Table 5. Since an outlier was identified in the old cemeterial group (highlighted in bold in the table), the value was excluded from the statistical analysis.

**Table 5:** BMD values (expressed as g/cm^2^) of the middle diaphysis for each sample. Median, mean, standard deviation (SD), minimum and maximum values are also reported for each group. Outliers are highlighted in bold. (*NA* middle diaphysis not available)

	SW			FW			OE			CM (old)			CM (young)		
	Sample	Sex	BMD	Sample	Sex	BMD	Sample	Sex	BMD	Sample	Sex	BMD	Sample	Sex	BMD
	SW 1	M	1.547	FW 1	M	1.639	OE 1	F	1.513	CM_o 1	F	1.127	CM_y 1	M	1.655
	SW 2	M	1.527	FW 2	F	1.495	OE 2	F	1.645	CM_o 2	M	0.876	CM_y 2	M	1.785
	SW 3	M	1.598	FW 3	M	1.321	OE 3	F	NA	CM_o 3	M	1.402	CM_y 3	M	1.760
	SW 4	M	1.702				OE 4	F	1.493	CM_o 4	F	1.130	CM_y 4	F	1.494
	SW 5	M	NA				OE 5	M	1.655	CM_o 5	M	1.323	CM_y 5	F	1.518
	SW 6	M	1.772				OE 6	M	1.583	**CM_o 6**	**M**	**1.679**	CM_y 6	F	1.481
	SW 7	M	1.574				OE 7	F	NA	CM_o 7	M	1.276	CM_y 7	F	1.608
	SW 8	M	1.763				OE 8	M	1.729	CM_o 8	F	1.217	CM_y 8	M	1.560
	SW 9	M	1.424				OE 9	M	1.672	CM_o 9	M	NA	CM_y 9	F	1.456
	SW 10	M	1.332				OE 10	F	NA	CM_o 10	F	0.978	CM_y 10	M	1.665
	SW 11	M	1.536				OE 11	F	NA						
	SW 12	M	1.673												
	SW 13	M	1.689												
	SW 14	M	1.481												
	SW 15	M	1.564												
**Median**			1.569			1.485			1.645			1.174			1.584
**Mean**			1.584			1.495			1.613			1.166			1.598
**SD**			0.126			0.159			0.087			0.176			0.116
**Min**			1.332			1.321			1.493			0.876			1.456
**Max**			1.772			1.639			1.729			1.402			1.785

BMD analysis showed comparable results between the sea water and the outdoor samples (Table 5). In both cases, BMD values ranged from 1.332 to 1.772 g/cm^2^, with a mean ± standard deviation (SD) of 1.6 ± 0.1 g/cm^2^. Similar findings were also detected on the samples from fresh water environments. Even if few samples were available in this case and statistics could not be considered, the three samples showed values comparable to the previous groups.

Differently, lower values were observed in the CM_old sample, whose bone mineral density ranged from 0.876 to 1.402 g/cm^2^, with a mean ± SD of 1.166 ± 0.176 g/cm^2^. In contrast to the old cemeterial group, femur samples from younger individuals coming from the same burial context (burial in coffin and a PMI of 20 years) showed a bone density similar to the first two groups (SW and OE groups) (mean, 1.598 g/cm^2^; SD, 0.116 g/cm^2^; min, 1.456 g/cm^2^; max, 1.785 g/cm^2^), highlighting again differences with the older subjects of the entire sample (Table 5).

These observations were corroborated by statistical analyses. ANOVA test showed a significant difference among the four major groups (p value < 0.001), and specifically significant differences were observed between the old cemeterial group with the sea water, outdoor and young cemeterial samples (p values < 0.001 in all cases by pairwise comparisons carried out using the Tukey HSD test).

**Fig. 4:**
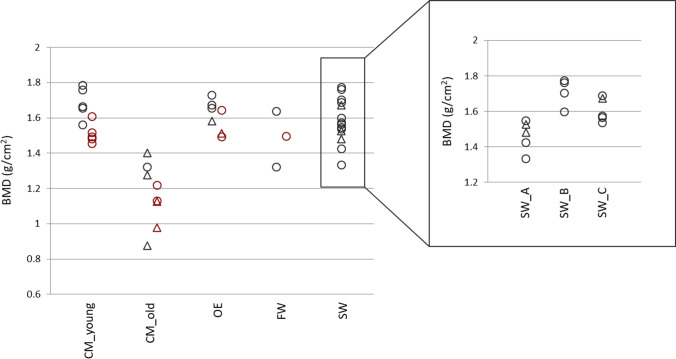
BMD values grouped by sex, bone and environment. Data for each environment are reported (CM, buried in coffin; OE, outdoor environment; FW, fresh water environment; SW, sea water environment). Femur and tibia samples are showed as circle and triangle, respectively, while male and female samples are in black on the left and in red on the right columns of each group. In the box on the right, sea water samples are divided by position in the boat (A, in the sea bottom; B, in the deck; C, in the cargo)

Considering the samples depending on the sex of the individual and the bone selected for the analysis (femur or tibia), additional information was pointed out. Figure 4 reports BMD values for each group of samples, distinguishing among males and females, as well as the bone sample (femur and tibia) and environments.

Female samples were generally characterised by lower BMD values compared to the males of the same group. A similar trend was observed between bone samples especially in the CM_old sample, where femurs showed greater values than tibiae.

Looking at BMD values distribution, the trend described before was more evident. Specifically, the majority of BMD values were in the range between 1.4 and 1.8 g/cm^2^ for both the aquatic and outdoor environments and the CM_young samples, while values lower than 1.4 g/cm^2^ were recorded in the CM_old sample (between 0.8 and 1.4 g/cm^2^). A similar scenario was outlined when comparisons were performed among the only male groups, even if the CM_young and the outdoor samples showed values only greater than 1.6 g/cm^2^. To this purpose, a deep analysis of the sea water group highlighted that BMD values between 1.4 and 1.6 g/cm^2^ were observed especially in the A and slightly in the C groups (i.e. the samples recovered in the sea bottom and cargo, respectively), whereas greater values were recorded in the B samples (i.e. samples recovered in the deck of the ship).

## Discussions

The present pilot study provided a preliminary insight on the state of preservation of bodies and bone samples from a marine environment, and specifically from great depths (400 m) and with short PMI (4–14 months). This was done from several points of view (macroscopic, microscopic and densitometric), by comparing data of similar samples collected from individuals which were buried in coffins, as well as recovered in outdoor or fresh water environments.

Macroscopic and microscopic methods were selected as they are the reference methods in bone taphonomy research and the most used to describe bone tissue preservation [see 8, 21, 26, 45–48]. However, to collect data mainly on the mineral component, dual energy X-ray absorptiometry was selected as non-destructive method able to measure the mineral content and to provide additional data before carrying out any further destructive analyses.

In literature, the few studies focusing on marine taphonomy are limited to body decomposition [24–27] and to experimental investigations on pigs or bioerosion [28–34]. However, not many analyses were carried out on the skeletal tissue in order to evaluate the cellular and molecular changes which occur in marine contexts in contemporary samples.

Our results showed an overall good preservation of bone tissue of the sea water samples from both the macroscopic and microscopic point of view, regardless of the state of decomposition of the body. Only three samples displayed cortical bone erosion possibly due to fauna activity in agreement with the observations performed by Pokines et al. [26] and the experiments carried out using pig carcasses by Anderson and Bell [32–34]. Despite bone loss, the samples were characterised by well-preserved tissue, and no microorganism invasion in the form of tunnels such as those described in several studies [8, 19, 20, 23, 35] was detected. The absence of such microbial activity could be explained with an insufficient time since death to allow microorganisms’ invasion and growth. The observations performed by Yoshino and colleagues [19] supported this theory. In fact, they observed microscopically the first signs of microorganism activity in recent human remains recovered from the marine context 4–5 years after death. Similarly, even if using a different substrate (limestone) and focusing on macrobioerosion, Färber and colleagues [31] (experimental condition: close to Greek cliff, water depths between 3 and 17 m) observed no activity within the first year of exposure, thus identifying an increasing diversity and intensity of bioerosion agents over time. However, time could not be the only variable. In fact, since some archaeological samples (tenth to sixteenth centuries) showed bone matrix characteristics similar to those observed in fresh bones [20, 35, 36], environmental conditions have to be also included among the factors influencing bone tissue preservation. Several studies highlighted the effects of environmental parameters such as temperature, light, depth, dissolved oxygen on faunal compositions and diversity related to body preservation [24, 25, 28–34]. Generally, a decrease in life and diversity has been observed with increasing depths and differences were recorded in different seasons, with the lowest activity and diversity documented in winter at the deepest depth considered (250 m) [30]. In their study Arnaud and colleagues [35] explained the good preservation of the observed bone matrix as the result of a wet salting procedure of the sample during the post-mortem period. In such a case, the salt contained in the sea water acts similarly to the salt-fish conservation, as it penetrates into the tissue with a better bone structure preservation.

In order to verify the presence of differences among bones coming from different environments, seawater samples were compared with other cases coming from outdoor and fresh water environments and cases buried in a coffin. Previous studies in literature compared samples from different post depositional contexts [15, 19, 21, 23], but none of them investigated taphonomic parameters such as the general body preservation, the macroscopic and microscopic tissue appearance and the bone mineral content.

When comparing samples from the various analysed contexts (sea water, fresh water, outdoor and burial in coffin), no differences were observed in terms of bone macroscopic and microscopic preservation. Specifically, the microscopic aspect proved similar high OHI scores in both undecalcified and decalcified thin sections, suggesting a good preservation of both organic and inorganic components. Only few samples from individuals buried in coffins showed limited microorganism invasion (10–30%). Regardless the bone element analysed, the present results are in agreement with those reported in literature: limited microorganism invasions were found at mid-diaphysis in femurs and tibiae of buried individuals by Caruso et al. [48], and the appearance of first signs of microbial activity within the bone tissue was found near the surgical neck of humeri after 15 years of decomposition on the ground by Yoshino et al. [19]. The similar preservation observed suggested that regardless the region of sampling, bone diaphysis shows a comparable bone tissue preservation. Differences were instead highlighted on the state of preservation of the body. In fact, an advanced decomposition was found mainly in fresh water and outdoor samples, while a greater amount of seawater and buried cases were partially or completely skeletonised. The complete skeletonization of samples from buried corpses could be ascribable mainly to the longer time since death (PMI: 20 years); for this reason, a higher number of samples coming from a similar environment and with a shorter PMI (1 year) are needed to perform additional comparisons. On the contrary, samples recovered from the sea showed results at times slightly opposite to what has been reported in literature: Ellingham et al. [27] discoursed about a decomposition process slower than that occurring on ground for corpses decomposing in seawater contexts because of the lower temperatures, the absence of insects and the salinity which decreases bacterial activities. However, as highlighted by Anderson and Bell [34], at depths of 100–300 m remains are mainly scavenged than decomposed, and this data might justify why the majority of bodies proved partially or completely skeletonised in our sample. In this regard, faunal composition and necrophagous communities are the main factors influencing the speed of skeletonization of a body [24]. Examples of skeletonised and disarticulated bodies with a short PMI (few months) have been described in literature [24, 32].

Even if slight differences were highlighted among the groups, body preservation seemed not to influence the macroscopic and microscopic appearance of each sample. In fact, even in case of complete skeletonization of the body, macroscopic and microscopic bone appearance was similar in all the contexts. Similarly, no relation exists between macroscopic and microscopic preservation, as highlighted within the small fraction of samples showing bone weathering on the surface, but a well-preserved bone tissue microscopically. In addition, the features analysed in our study seem not to be predictive of the depositional context, both when samples with comparable and different PMIs were considered. The only exception seems to be the peculiar pattern identified in the sea and fresh water samples (blackish/darkened areas). However, such a pattern is difficult to interpret and the origin and causes are still unknown. To this purpose, additional investigations are needed by high-resolution technologies, such as backscattered electron imaging in a scanning electron microscope (BSE-SEM), already used in taphonomic studies of bone tissue [10, 15, 21, 23, 51].

Densitometric radiological investigation provided interesting data to taphonomic research. To date, studies focused especially on bone portion “survival” without checking whether differences exist between environments. Therefore, this is the first study in which different depositional contexts are compared and where data on sea water samples are reported.

Differently to anthropological analyses, densitometric radiological investigation highlighted a significant difference between sea water and outdoor samples with old cemeterial cases. The lower values observed in the latter can depend on several variables, among which the environment, age at death, pathology or the bony part we analysed. In fact, the old cemeterial group was the sample mostly represented by tibiae (50% of the sample compared to the 18–27% of the other groups) where the BMD values found were clearly lower than those observed on femurs. Because it was difficult to verify all the variables and especially considering the decrease of the mineral content in the old population already proved in literature [52–54], we decided to select ten individuals from the same environment (burial in coffin) whose age at death and kind of bone were comparable with the outdoor and sea water samples (age range: 20–43 years old, bone: femur). The obtained BMD values at the middle diaphysis ranged between 1.456 and 1.785 g/cm^2^ (mean: 1.598 g/cm^2^, SD: 0.116 g/cm^2^), and no differences were observed when comparing these values to those obtained from sea water samples and outdoor cases, excluding any difference among these environments. When fresh water samples were also considered, even if in a small number, similarities were observed with the majority of the samples, highlighting once more the difference with the old group.

Since the sea water samples were all collected from male individuals and considering the well-known differences in bone mineral density existing between males and females [37, 52], comparisons were also carried out limiting the analyses to the only male fraction. Also for this sub-analysis we found similar conclusions, even if cemeterial (young individual), and outdoor samples showed values slightly greater than sea water bones. These variations may be related to the reduced number of samples and/or the position of bodies within the ship. In support to the second hypothesis, BMD values recorded suggested a decrease of bone mineral density in samples collected from individuals recovered in the sea bottom and cargo, while greater values were observed in samples recovered from the deck. However, sample size has to be increased in order to check this variation and to identify the variables involved.

The present study has some limitations. Some samples, especially those buried in coffins, had a PMI longer than the other cases, and no data were available 1 year after death. However, since a well-preserved bone tissue has been macroscopically and microscopically observed in the majority of the cases, we could assume a bone tissue preservation comparable to the other samples even in the early post-mortem period. Concerning bone densitometric investigation, no information was available about BMD at the time of death, preventing to carry out a longitudinal analysis for this parameter assessing its possible changes over the time in each environment. Therefore, we focused mainly on the data after the recovery in the several depositional contexts in order to verify if any difference exists and if BMD can be considered a relevant predictive marker of the depositional environment in forensic cases. An additional limitation lies in the unequal number of samples from each post depositional contexts and especially the small size of the fresh water sample. This requires in the future further analyses on larger sample groups in order to verify the observations reported. Nevertheless, even if no additional data are available, the present study would provide an initial overview of the state of preservation of the bone tissue comparing samples with different post-mortem histories and by adding radiological densitometric methods to the common anthropological investigations used in bone taphonomy research.

## Conclusion

The present pilot study represents a first taphonomic attempt to compare samples of contemporary human remains recovered from different environments and PMIs by several perspectives, enriching literature on body and bone preservation, from a macro-, microscopic and radiological point of view. In particular, since no studies are available concerning contemporary human remains recovered from the sea at greater depths such as 400 m and especially in the Mediterranean Sea, these observations, even if on a small sample, provided further data on marine bone taphonomy. The analyses performed highlighted a similar preservation of the bone tissue in the four environments, both macroscopically and microscopically, despite the state of preservation of the body. Similar conclusions were outlined by radiological densitometric investigations, showing no differences among the contexts. Further analyses need to be performed using higher resolution technologies, larger sample sizes, human remains with different PMIs and recovered from various environmental contexts, in order to possibly highlight changes and differences for longer period of times and to obtain a more complete taphonomic picture.

In the meantime, the results obtained in this study suggest that no differences exist among the four contexts at the time of recovery and that neither the bone preservation nor the mineral content/density can be considered predictive of the depositional environment, especially for cases with PMI shorter than 14 months.

## Data Availability

All data analysed during this study are included in this published article
